# Supplementary material for: Light Regulation of LoCOP1 and Its Role in Floral Scent Biosynthesis in Lilium ‘Siberia’
Source: Plants (Basel). 2023 May 16;12(10):2004. doi: 10.3390/plants12102004 (PMC10223427; doi:10.3390/plants12102004)
Supplement: Supplementary file 1 [file plants-12-02004-s001.zip › plants-2387352-supplementary material.pdf]

Supplementary Material

Table S1. *LoCOP1* ORF nucleotide sequence

LoCOP1 ORF nucleotide sequence
ATGGCGGACGCCTCCGTCGGCGCTCTCGTCCCGTCTGTGGGAAAGCCCGAGGCCCG<br>CTCCTCGATGCCGGCGGTTTCCTCGTCGGAGACGACCGACACTGCCGCCGTGATGG<br>CGGTAGATAGAGTGAGACCCAGAGCATCGGCGGCGCCGGCGGAAGAAGAAGAGGA<br>GCAGGACAAGGATTTGCTGTGTCCGATCTGTATGGGGACGATTAAGGACGCGTTTCT<br>GACGGCGTGCGGGCACAGCTTCTGCTACATGTGTATTGTCACGCATCTTCAGAATAA<br>GAGCGATTGTCCGTGCTGTGCTCATTATCTCACGAAGAATAACATATTCCCCAATTTC<br>TTGCTCAATAAGCTTTTGAAGAAAATGTCTGCCCATCAAATTGCTAAAACTGCATCA<br>CCGGTTGAGCATCTCCGTCTGGCATTGCAACAGGGTTGTGAAATGTCGATTAAGGAG<br>ATGGACAGTTTGCTATCTCTGCTTACAGAGAAAAAGAGGAAGATAGAGCAACAAGA<br>GGCTGAGACTAACATGCAGATTTTGCTTGATTTCCTGCACTGTCTTCGGAAGCAAAA<br>GCTAGAGGAGCTAAATGAGGTTCAAAATGATCTGCAATATATCAAAGAAGATGTGAT<br>TGCTGCAGAGAAACACAGGATAGAGTTATATCGAGCAAGGGAGAGATACTCTTTAA<br>AATTGAGGATGCTTTTGGATGATTCTTTGGCAACAAAGTTATGGCCTTCCACACTTGA<br>GAAGCACAATAATATCCTTGTCCCCAATGTTCGTAGCTCATATGGCGGGACATCTTCC<br>GGAAATTTCCAGACTCAAAAAGTTGATAACAAAGCTCAAGGAAGCTATCCAGGGCA<br>GCAGAGGAAAGATGCATTTAGTGGTTCAGATTCACAACAATCTCTTATGCAATCAGG<br>GCTGGCTGTAGCTAGAAAGAGACGAGTACATCAACAGTTTAATGAGCTACAAGAGT<br>GCTACTTACAAAAACGGCGCCTAGGAGCCAACCAACAATGCAGGCAAGAAGGAGA<br>TACACGTTCTACAAACAGAGAAGGCTATCATGCAGGTCTTGAGGATTTTCAGTCCGT<br>ACTGACTACCTTTACTCGATACAGTCGATTGCGTGTCATTGCGGAACTTAGACACGG<br>GGATCTCTTTCACACTGCAAATATTGTATCCAGCATAGAATTCGACCGTGATGACGAG<br>TTATTTGCTACTGCTGGAGTTTCCAAACGGATAAAAGTTTTTGAGTTCTCCACAGTAA<br>GCTCGGTTGTAAATGAGCCTGCCGAGGTACACTGCCCTGTTGTAGAGATGTCTACCC<br>GATCTAAACTTAGTTGCTTAAGTTGGAACAAGTACTCGAAAAACATCATTGCAAGTA<br>GTGATTATGAAGGCATAGTGACTGTTTGGGATGTAAATACTCGCCAGAGTGTGATGG<br>AATACGAAGAGCATGAAAAGAGAGCATGGAGTGTTGATTTTTCACGCACAGAACCA<br>ACCATGCTGGTATCAGGTAGCGATGATTGTAAGGTCAAAGTTTGGTGCACAAAGCAA<br>GAAGCTAGCGTGTTCAATATCGATATGAAAGCAAATATATGTTGTGTCAAATATAATCC<br>TGGATCCAGCTTTCATGTGGCGGTTGGTTCTGCTGATCACCACATTCACTATTTTGAT<br>TTGAGAAATACCAGTGCTCCACTCCATGTCTTCAGAGGTCACAGGAAAACAGTTTC<br>GTACGTCAAATTTCTATCAACTAATGAACTTGCATCTGCATCCACGGACAGTACATTG<br>CGCTTGTGGGATGTAAAAGACAACTGTGCTGTTCGAACATTCAAAGGCCACGCGAA<br>TGAGAAGAATTTCGTAGGTCTGACAGTGAATGACGAGTATCTCGCATGTGGCAGCGA<br>AACAAATGAAGTCTTTGTGTACCATAAGGCCATCTCGAAGCCGTCAACGTGGCATCG<br>ATTCAGTTCCTCCGAACCGGACGTTACTGATGATGATGCTGGATCATACTTCATAAGT<br>GCTGTATGCTGGAAGAGTGACAGCCCCACGATGTTAACTGCAAACAGTCAGGGAAC<br>AATCAAGGTCCTTGTGCTTGCTGCCTGA

Table S2. LoSAP ORF nucleotide sequence

LoSAP ORF nucleotide sequence
ATGATACCTAACTCGTCCGCAGGCGCCAGCTCCACCGTGCGAGTGGAGCCGAGAGGTCTCTGGCCT
AATCTCACCAGGCTAGGCGGCGCGCTGCCAAATTCGTTAGGTAACAGACCTCCACTAACTATGGAA
ACTAGAGATGATGCATGGGGTTTATTTCTTCCGCAGATTGGGGCTCGCCGGCCGCCTCTCCCAATCC
GGACGAGTACTCCGCCTCCGTCTGGGTTCCCACAATTCGCAATCAAGAAGGGGAAGGAGGTTGCAT
CTGCCTCCCGGATTCAGAGTAGTAGGACGGAGAAGAACCAGAGTACGGAGATTGCTTCTAATTTGC
CTCATCGGCAAGGTGGGAAACCAGTGTTTGGCGGTCGGGGAGGGAATGCGAGTGTTCCCGACCCT
CATCATGAGGGTACCAGCATGAGGGAGTGGCTGAAACAGAGGCAGCACAAGTTCAATAAGGACGA
GAGGCTCACCTTTTTCCGGCAGATATTGGAGCTGGTTGAAATTACACATTCACAGGGGCTGTCGCTG
CATAGTTTGAGACCGTCTTATTTCATGATATTCCCGATGAATCAGATCAAGTATATTGGTTCCTTGGTT
CCGGAAGGGCAGCCGAGGCCGCTGACGCGACCGGCAGACGAAGATGGTCGCTCTTCGGAGAGCA
GTGGTTCAAAGAGGAAGATGAATTTGCAACCAGAGGCAGTGGGTAATTTATCACTGAAGCGCCGA
AAAGTCTGGCAACCTGATTATGGGACTTTGACGGCACAAATTTCTGACAATGGCCATCGGAGACAA
CATCTTGAACTGGGTCCCGATCACAATTTCCAGATTCCAGCTATACAGTTGTTGAAATCTGATATTAG
GAAAATGGAGGAGAGATGGTATGCCAGTCCTGAGGAGCAAAATGACAACATATGCTATCTTTCTTC
AAATATCTATAATCTCGGTGTTCTTGTTTTCGAGCTCTTTTGTCATTTCGACACTTTGGAAGCACATTA
TGCTGCAATGTCCGATCTGCGGCATCGGATTCTCCCGCCAAATTTCTTATCAGAAAATCCCAAGGAG
GCTAGTTTATGTCTTTGGTTGCTCCATCCAGACCCTTCTTCTCGACCCAAGTCAAGGGACATTTTAAC
ATGTGGCCTAGTATGTGAAGGGAGAAACCGGTTGTCGCTAAATCAGTCATCAACATCAATTGATGAA
GAGGATGCAGAGGCAGGCTTATTACTGCACTTCCTATTTACCTCAAAAGACCAGAAAGAGAAGGAT
GCTGCTAAGTTAGTAGCAGATCTTGGTAGCATACAAGCGGACATTGTGGAGGCTGAGAAAAGACAC
TCATCAAGGGTCAAGACTCTTTCTAATGCCAGAGATTTGTTGACCAACTCCATTGAAAATCCAGATA
TAAGTCTTCACAGAGGACTAATACATGAAAAGAATGTGTCTCAGGTGTCCATATCAAGTACACAGA
ATGATAGGTTGATGATTAATATTGACCAACTAAAAAATGCATACTTTTCTATGAGATCTGAGATTGAG
CTCTCTGAAAGTAATGTTCCAGTACGTTCAGATATAGATGTTCTAAAGATCCGTGATAGAAACTTTCA
AGTCCGAAGTGATACCAATTCATTGAAGGAATCAACTGATCGTTTAGGTACCTTCTTTGAAGGTTTG
TGCAAGTATGCTCGATATAGTAGATTTGAAGTATGCAGCACCCTAAGGAATGTGGATATCCTCAACTC
GTCAAATGTCATTTGCTCTTTGAGTTTTGACCGGGATGAAGACTACTTTGCTGCTGCAGGAGTTTCA
AAGAAGATACAAATCTTTGAATACAGTTCTCTTTTGAATGACAGTGTCGATATTCATTATCCATTGAT
TGAGATGTCAAATAGATCCATGCTCAGTTGTGTCTGCTGGAATAATTACATCAAGAACTATTTAGCTT
CAACTGATTATGAAGGTGTGGTTCAGTTATGGGATGCAAGCACCGGTCAAGGATTCACAAAGTACA
CAGAACATCAAAAGAGAGCTTGGTCCGTCGATTTTTCTCAGACGGATCCAATGAAGTTGGCTAGTG
GAAGTGATGATTGTTCTGTGAAACTTTGGAGCATTAACGAGAATAGCTGTGTTGGTACAATCAGAA
GTATGGCCAACATATGTTGCGTGAAATTCTCCTCGTACTCCCATTTCTTGGCCTTTGGCTCTTCTGAC
TACAAAATATACTGTTATGATCTACGGATTACTAGAATCCCTTGGTGCACTTTGTCTGGACATGGAAA
GGCTGTCAGCTATGTGAAATTCATAGACCCTGAAACACTTGTTTCTGCATCAACTGACAACACACTC
AAGCTATGGGATCTTAACAAGACAAACTCAGGTGGATTGTCCACTGATGCCTGCAGCTTGACCTTC
AGCGGTCATACAAACGAGAAGAATTTCGTGGGTTTATCTGTTTCTGATGGATACATAGCATGCGGTT
CAGAAACCAATGAAGTTTATACTTATTATAAAACTTTACCCATGCCAATCACTACTTACAAATTTGGT
TCCGCTGATCCAATTACTGGAAAAGAGATGGGCAACGACAACAGACAATCTGTTTCAAGTGTATGC
TGGAGAACCAAGTCAAACACTCTTGTCGCGGGGACCTCCAATGGGAGCATGAAACTGCTGAAGTT
GGTCTAG

Table S3. Specific primer sequences used for gene clone, vector construction and quantitative real-time PCR.

Primer name	Sequence	Purpose
LoCOP1-F	ATGGCGGACGCCTCCGTC	*LoCOP1* gene clone
LoCOP1-R	TCAGGCAGCAAGCACAAGGAC	
GFP-LoCOP1-F	GGACTAGTATGGCGGACGCCTCCGTC	Subcellular localization
GFP-LoCOP1-F	CGAGCTCGGGGCAGCAAGCACAAG	
pGADT7-LoCOP1-F	TCCCCCGGGTATGGCGGACGCCTC	Y2H
pGADT7LoCOP1-R	CCCTCGAGCGGCAGCAAGCACAAGGAC	
VIGS-LoCOP1-F	AAGGAAGTTTAACGTAGCTCATATGGCGGGAC	VIGS
VIGS-LoCOP1-R	AACCACCACCACCGTCTCAAGACCTGCATGAT	
pOx-LoCOP1-F	GGGGTACCATGGCGGACGCCTC	Overexpression
pOx-LoCOP1-R	GGACTAGTAAGGCAGCAAGCACAAGGAC	
pUC-SPYCE-LoCOP1-F	GGACTAGTATGGCGGACGCCTCCGTC	BIFC
pUC-SPYCE-LoCOP1-R	GACGTCGACGGCAGCAAGCACAAG	
qPCR-LoCOP1-F	GTCCCCAATGTTCGTAGCTC	quantitative real-time PCR
qPCR-LoCOP1-R	GCTGCCCTGGATAGCTTCC	
GAPDH-F	GGTATTGTCGAGGGTTTGATG	
GAPDH-R	AGGGAGAACTTTGCCAACAGC	

Table S4. Gene IDs of *COP1* genes used for multiple sequence alignment and phylogenetic analysis.

Name	Species	Gene ID
OsCOP1	*Oryza sativa Japonica Group*	BAA94422.1
ObCOP1	*Oryza brachyantha*	XP_006647974.2
BdCOP1	*Brachypodium distachyon*	XP_010236060.1
TuCOP1	*Triticum urartu*	EMS63367.1
ZmCOP1	*Zea mays*	NP_001152482.1
SiCOP1	*Setaria italica*	XP_004954056.1
AcCOP1	*Ananas comosus*	OAY85246.1
CnCOP1	*Cocos nucifera*	KAG1327072.1
EgCOP1	*Elaeis guineensis*	XP_010913400.1
AsCOP1	*Apostasia shenzhenica*	PKA52651.1
PeCOP1	*Phalaenopsis equestris*	XP_020577071.1
DcCOP1	*Dendrobium catenatum*	XP_028554358.1
VvCOP1	*Vitis vinifera*	XP_002270330.2
SlCOP1	*Solanum lycopersicum*	NP_001234047.2
InCOP1	*Ipomoea nil*	AAG31173.1
GmCOP1	*Glycine max*	XP_003545597.1
GsCOP1	*Glycine soja*	KHN44754.1
CaCOP1	*Cicer arietinum*	XP_004491092.1
RcCOP1	*Ricinus communis*	XP_002534127.1
PtCOP1	*Populus trichocarpa*	XP_002321154.1
GrCOP1	*Gossypium raimondii*	KJB42844.1
BnCOP1	*Brassica napus*	NP_001302788.1
BoCOP1	*Brassica oleracea var. oleracea*	XP_013637215.1
AtCOP1	*Arabidopsis thaliana*	AT2G32950.1

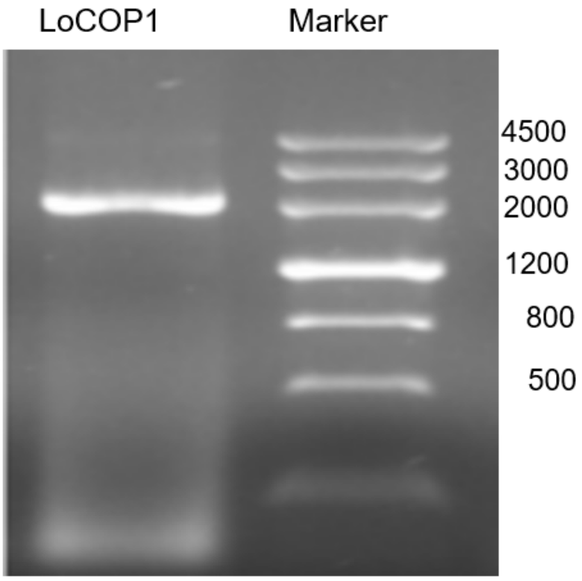

Figure S1. Electropherogram of a LoCOP1 clone

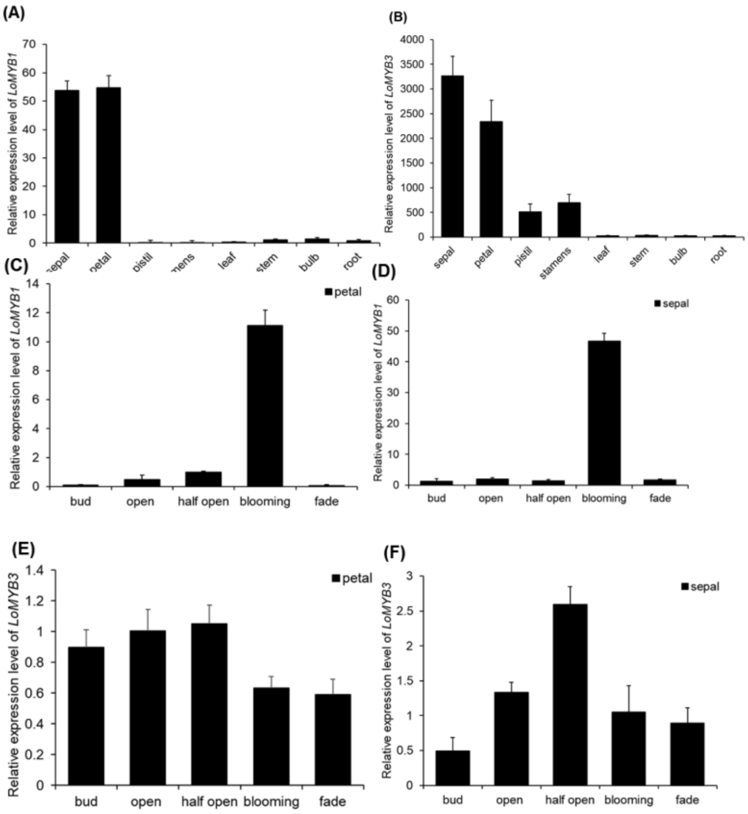

Figure S2. RT-qPCR analysis showing tissue-specific and development-regulated expression of LoMYB1 and LoMYB3. (A) Transcript levels of LoMYB1 in various tissues of Lilium ‘Siberia’. (B) Transcript levels of LoMYB3 in various tissues of Lilium ‘Siberia’. (C) LoMYB1 transcript

levels in the different development stages of petal. (D) LoMYB1 transcript levels in the different development stages of sepal. (E) LoMYB3 transcript levels in the different development stages of petal. (F) LoMYB3 transcript levels in the different development stages of sepal. Error bars show the standard deviations from tree biological replicates.

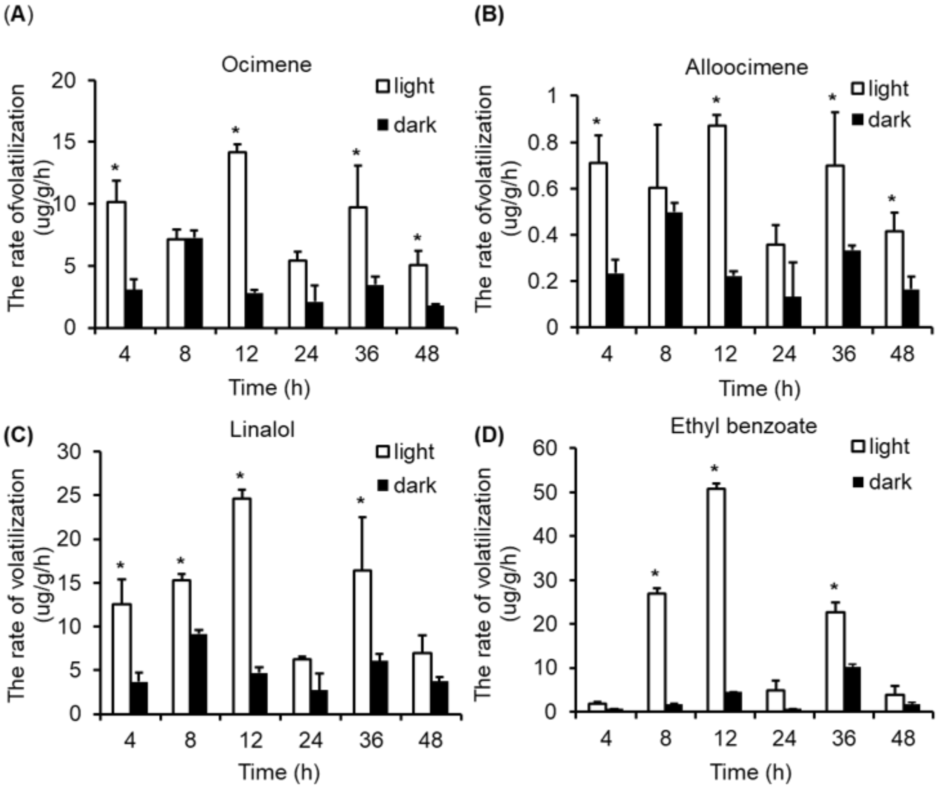

Figure S3. The rate of volatilization of the main floral fragrance components under continuous light and dark treatment. (A) The rate of volatilization of Ocimene. (B) The rate of volatilization of Alloocimene (C) The rate of volatilization of Linalol (D) The rate of volatilization of Ethyl benzoate. Asterisks indicate statistically differences (*p< 0.05). Error bars show the standard deviations from tree biological replicates.

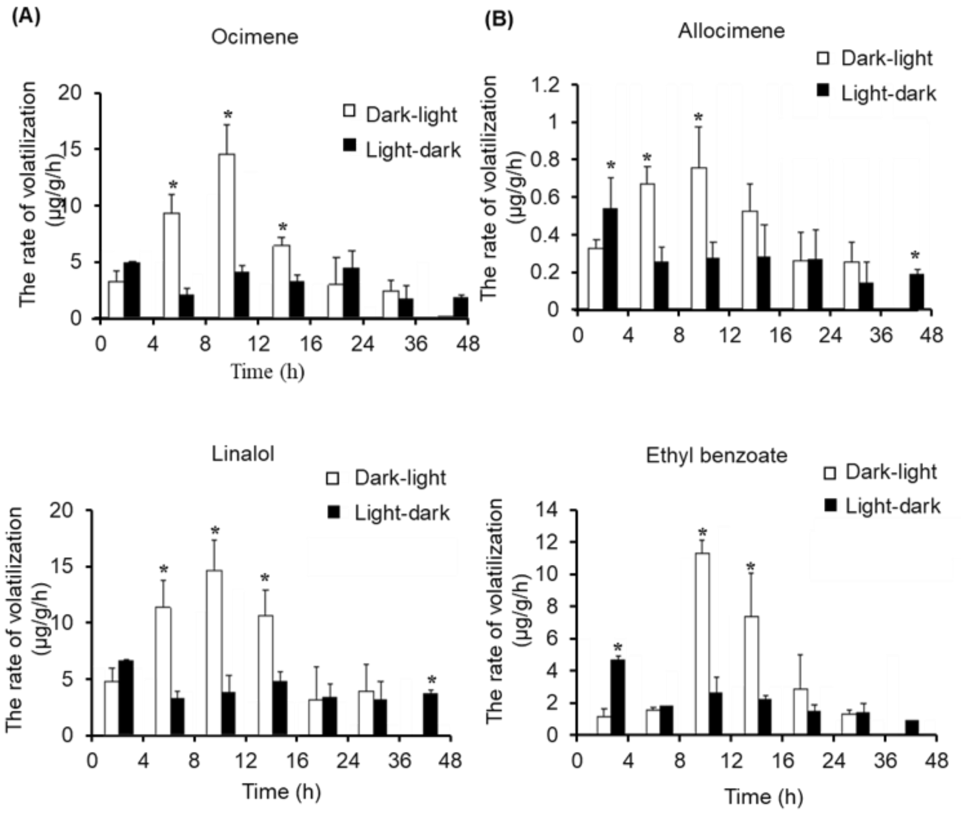

Figure S4. The rate of volatilization of the main floral fragrance components under light and dark transition. (A) The rate of volatilization of Ocimene at different time point after light and dark transition. (B) The rate of volatilization of Alloocimene at different time point after light and dark transition. (C) The rate of volatilization of Linalol at different time point after light and dark transition. (D) The rate of volatilization of Ethyl benzoate at different time point after light and dark transition. Asterisks indicate statistically differences (*$p < 0.05$). Error bars show the standard deviations from tree biological replicates.

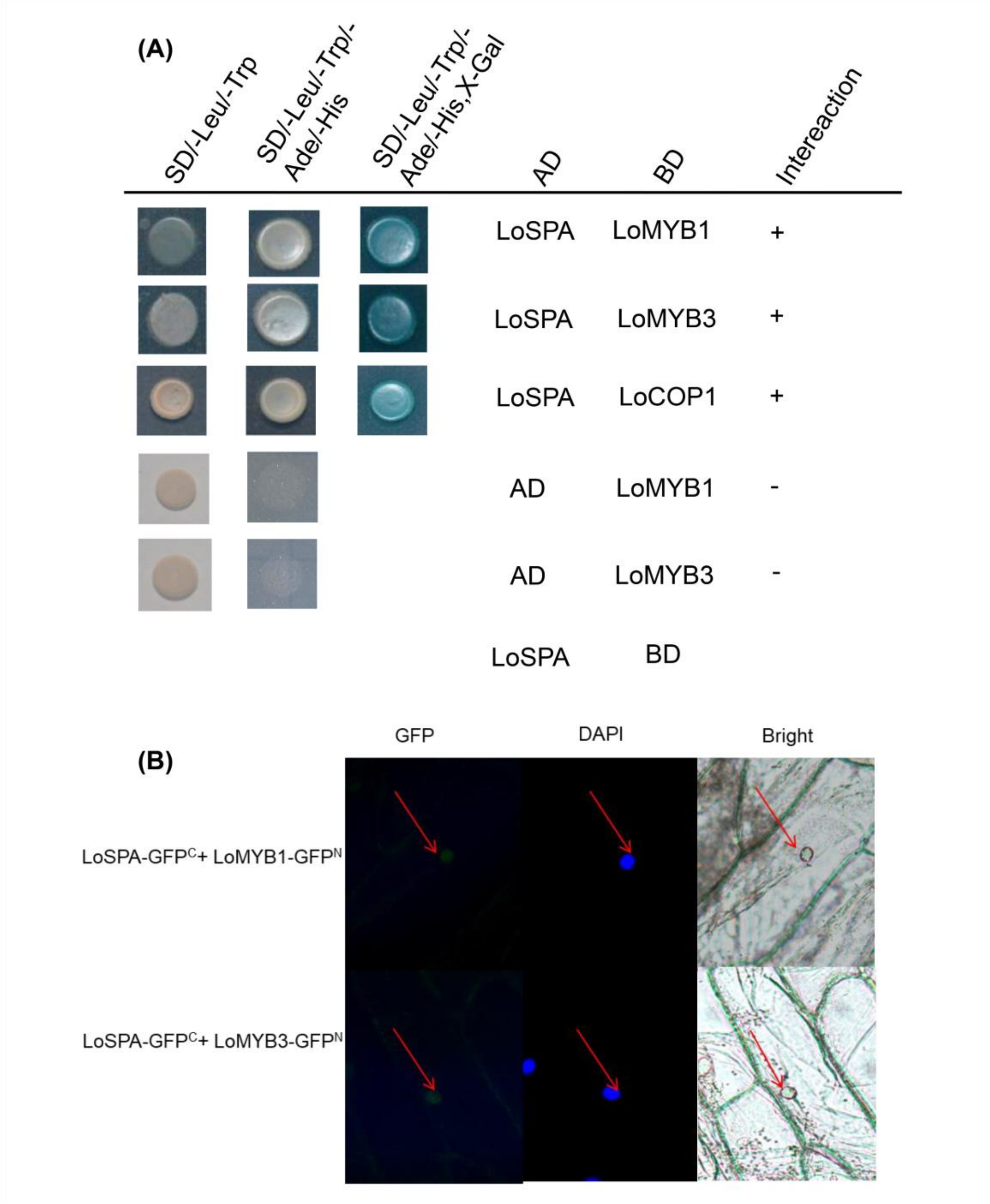

Figure S5. Interaction between LoSPA with LoCOP1, LoMYB1 and LoMYB3. (A) Yeast two-hybrid assays were conducted with selective growth combined with a 5-bromo-4-chloro-3-indolyl-b-d-galactopyranoside acid overlay assay. (B) BiFC interaction assays using onion epidermal cells. GFP fluorescence was detected at 2 d after transfection.

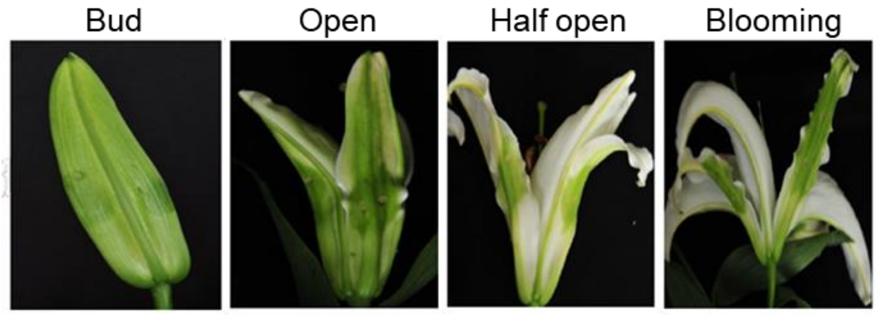

Figure S6. Growth and development condition of *Lilium* ‘Siberia’ flower after infecting by BMVS virus.
